# Intracerebral functional connectivity-guided neurofeedback as a putative rehabilitative intervention for ameliorating auditory-related dysfunctions

**DOI:** 10.3389/fpsyg.2014.01227

**Published:** 2014-10-29

**Authors:** Stefan Elmer, Lutz Jäncke

**Affiliations:** ^1^Division Neuropsychology, Institute of Psychology, University of ZurichZurich, Switzerland; ^2^Center for Integrative Human PhysiologyZurich, Switzerland; ^3^International Normal Aging and Plasticity Imaging CenterZurich, Switzerland; ^4^Research Unit for Plasticity and Learning of the Healthy Aging Brain, University of ZurichZurich, Switzerland; ^5^Dynamic of Healthy Aging, University Research Priority Program University of ZurichSwitzerland; ^6^Department of Special Education, King Abdulaziz UniversityJeddah, Saudi Arabia

**Keywords:** functional connectivity, EEG, neurofeedback, developmental dyslexia, rehabilitation, auditory-related cortex

## Abstract

Electroencephalography (EEG) constitutes one of the most eligible candidates for neurofeedback applications, principally due to its excellent temporal resolution best reflecting the natural dynamics of brain processes. In addition, EEG is easy to use and provides the opportunity for mobile applications. In the present opinion article, we pinpoint the advantages of using intracerebral functional connectivity (IFC) instead of quantitative scalp EEG for interventional applications. In fact, due to the convergence of multiple signals originating from different spatial locations and electrophysiological interactions, miscellaneous scalp signals are too unspecific for therapeutic neurofeedback applications. Otherwise, IFC opens novel perspectives for influencing brain activity in specific dysfunctional small- and large-scale neuronal networks with a reasonable spatial resolution. In the present article, we propose concrete interventional IFC applications that may be used to ameliorate auditory-related dysfunctions such as developmental dyslexia.

## FROM HISTORICAL BRAIN PERSPECTIVES TO MODERN NEUROSCIENTIFIC APPROACHES

During the 19th century, brain researchers took advantage of individuals suffering from brain lesions in order to determine the contribution of specific brain areas to different aspects of behavior, including perception, speech processing, motor skills, and cognitive functions (Zolamorgan, 1995; Leff, 2004). During the same century, an intellectual quarreling raged between researchers who believed that brain functions are localized in separable brain areas (localization view) and those who argued that the entire or parts of the cortex contributes to behavior (network view; for an historical overview see for example Leff, 2004). Nowadays, one can draw some hazardous analogies between modern neuroscientific approaches and historical perspectives on brain functions depending on the imaging technique used. In fact, functional magnetic resonance imaging (fMRI) has a very poor temporal resolution, leading to the illusory impression that specific brain functions are localized in distinct brain areas. Otherwise, due to the dynamic and blurred nature of electrical scalp signals (i.e., electroencephalography, EEG), one could naively come to the conclusion that the entire brain contributes to a specific behavior.

Currently, it is generally acknowledged that widely distributed, specialized, and dynamic cortical-subcortical networks form the fundamental basis of behavior (Bullmore and Sporns, 2009). Within this framework, EEG has gained more and more attention in the field of cognitive neuroscience, mainly due to its excellent temporal resolution (in the range of milliseconds) enabling to capture the dynamic dimension of brain functioning in a more realistic manner than neuroimaging does. In addition, based on novel mathematical applications it is now possible to estimate the intracerebral origin of scalp signals (Scherg, 1990; Pascual-Marqui et al., 1994) as well as to objectify intracerebral functional connectivity (IFC) in real-time (Canuet et al., 2011; Kühnis et al., 2014) with a reasonable spatial resolution (Pascual-Marqui et al., 1994; Phillips et al., 2002). Thus, EEG is particularly suitable for comprehending the dynamic interplay between specific brain regions within local and global neuronal networks in both natural and dysfunctional brain conditions.

## NEURONAL NETWORKS: THE BEARING SKELETON OF BRAIN FUNCTIONS

Currently, there is no doubt that cognition (Langer et al., 2013), motor functions (Jin et al., 2012), and perception (Wu et al., 2012) do not function in isolation but are embedded in neuronal assemblies consisting of networks influencing each other’s through excitatory and inhibitory signals (Fell and Axmacher, 2011). Such small- and large-scale neuronal networks can be represented by using both functional and structural data as well as by taking into account different parameters, like white matter integrity (Hagmann et al., 2007; Elmer et al., accepted), cortical thickness (Hanggi et al., 2011), cortical surface area or volume (Bonilha et al., 2004; Sanabria-Diaz et al., 2010), hemodynamic responses (Rehme et al., 2013), or even intracerebral oscillatory phase synchronization values (Langer et al., 2012; Kühnis et al., 2014). Such neuronal networks can for example be modeled by taking into account mathematical graph theories (i.e., small-world networks) where most nodes within a network can be reached from every other by a small number of steps. This implies that efficient systems with small-world topology are characterized by a high local clustering coefficient (i.e., the degree to which nodes in a graph tend to cluster together) and short path lengths between distant nodes (Bullmore and Sporns, 2009). The advantage of focusing on such networks rather than on localized brain characteristics is that the former can support both segregated/specialized as well as distributed/integrated information processing (Bullmore and Sporns, 2009).

From a functional perspective, it is assumed that brain regions that do the same at the same time are somehow interconnected (i.e., functional connectivity). Functional connectivity and systemic brain organization can be described by using dynamic causal modeling (Eickhoff et al., 2009), Granger causality (Jancke, 2012), or correlative analyses between brain signals (for example signal amplitude, current density, power, or phase synchronization) originating from different spatial locations (Langer et al., 2012). Even though it results evident that structural and functional brain properties are mutually related (Miranda-Dominguez et al., 2014), the advantage of focussing on functional connectivity is that it enables to capture the dynamic nature of the human brain in different time-scales, ranging from milliseconds (i.e., EEG) to several seconds (i.e., fMRI).

## EEG AND INTRACEREBRAL FUNCTIONAL CONNECTIVITY

The discovery of EEG by Berger (1929) can be considered as one of the most important historical breakthrough in the field of neurology and cognitive neuroscience. This specific technique builds up on single electrodes that are fixed on the surface of the scalp for recording electrical brain activity. Through different applications in the field of electrical engineering (i.e., signal amplification, impedance reduction, etc.), it became possible to measure the summed electrical postsynaptic activity that is locked or unlocked to an external (for example auditory stimulation), or internal (for example imagery) event at the surface of the scalp. Such electrical brain activity can be quantified, for example, by evaluating the amplitude and timing of event-related potentials (ERPs), power spectra in different frequency ranges over time, or the degree of phase alignment (i.e., coherence) in a specific frequency band between single scalp electrodes.

In the last 15 years, novel mathematical applications render it possible to overcome the so called “inverse problem” of intracerebral EEG source estimation (Pascual-Marqui et al., 1994) however, with some drawbacks in terms of spatial resolutions (Phillips et al., 2002). Currently, several toolboxes and software (http://en.wikipedia.org/wiki/Comparison_of_neurofeedback_software) can be used for estimating intracerebral brain activity based on the electrical signal recorder from the surface of the scalp. These new technologies imply that for each signal measured on the surface of the scalp it becomes possible to estimate intracerebral brain activity for each voxel, Brodmann area, or region of interest (ROI) in the form of current-, or spectral-power density by retaining phase information. Therefore, all these measures can be taken for modeling IFC networks (see previous section).

**FIGURE 1:**
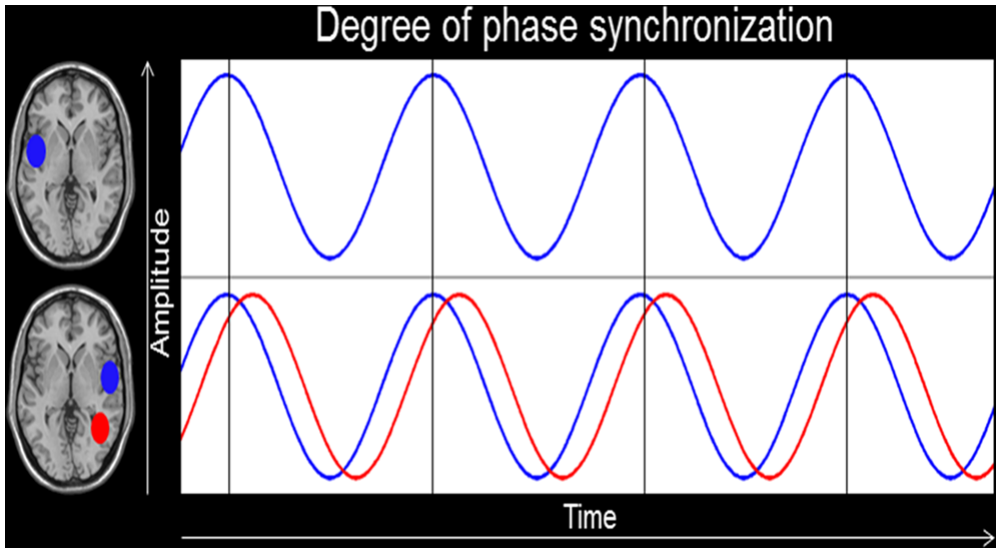
**Intracerebral functional connectivity (IFC).** This figure depicts the degree of phase alignment between two regions of interests (ROIs) in the left and right hemisphere. The bottom blue oscillation provides an example of perfect phase synchronization, whereas the red one shows a temporal lag in phase synchronicity.

In turn, we will provide two examples of practical applications of IFC in a specific group of experts, namely professional musicians. In a first study, we measured professional musicians and non-musicians by using EEG and IFC analyses. We postulated that auditory-specialization (Elmer et al., 2012; Marie et al., 2012; Kuhnis et al., 2013) and asymmetry (Schneider et al., 2002) in musicians should be dependent, at least in part, by the amount of interhemispheric communication between the left and right auditory-related cortex (ARC). Based on this assumption, we measured intracerebral phase synchronization (see **Figure 1**) in the theta, alpha, and beta frequency range between the two ARC in musicians and non-musicians. We found support for our hypothesis in that musicians showed increased IFC between the two ARC as well as a relationship between IFC and the amplitude of auditory-evoked potentials (Kühnis et al., 2014).

A second example that depicts a relationship between IFC and expertise arises from a recent study of our group (Elmer et al., under revision) where we tried to integrate two apparently opposite perspectives on absolute pitch, that is the ability to recognize the chroma (i.e., pitch) of a tone without a reference tone (Levitin and Rogers, 2005). In this context, some researchers argue that this specific ability relies on an optimized “early categorical perception” at the processing level of the left ARC (i.e., perception; Siegel, 1974), whereas others suggest that the distinctive trait of AP more likely derives from mnemonic facilitation (Elmer et al., 2013) enabling “pitch labeling” mechanisms by recruiting left-sided prefrontal brain regions (i.e., cognition; Zatorre et al., 1998). By combining EEG and resting-state IFC, we evaluated phase synchronization between the left ARC and the left dorsolateral prefrontal cortex in the theta (∼ 4–7 Hz) frequency range in a group of musicians with and without AP. Theta oscillations have previously been shown to reliably reflect mnemonic processes (Kahana et al., 1999; Caplan et al., 2001; Ward, 2003; Sauseng et al., 2005), information integration (Ward, 2003), and neuronal communication between distinct brain regions over long-range circuits (Ward, 2003; Polania et al., 2012). Results revealed that in AP musicians perceptual and cognitive subdivisions of the human brain are tightly coupled through oscillatory theta phase-alignment. In addition, within the AP group this specific electrophysiological marker was predictive of pitch-labeling performance by explaining about 30% of behavioral variance. These two EEG studies target at illustrating practical applications of IFC analyses for evaluating systemic brain reorganizations rather than focusing on localized brain functions in isolation. This point of view is also supported by a recent paper of Seither-Preisler et al. (2014) providing specific evidence for increased bilateral synchrony of the primary auditory evoked responses collected at the surface of the scalp in children undergoing musical training compared to children suffering from attention-deficit hyperactivity disorder. Interestingly, this functional dysalignment of auditory-evoked brain responses was accompanied by anatomical specificities of auditory-related brain regions.

## THE BASIC PRINCIPLES OF INTRACEREBRAL FUNCTIONAL CONNECTIVITY-GUIDED NEUROFEEDBACK

**FIGURE 2:**
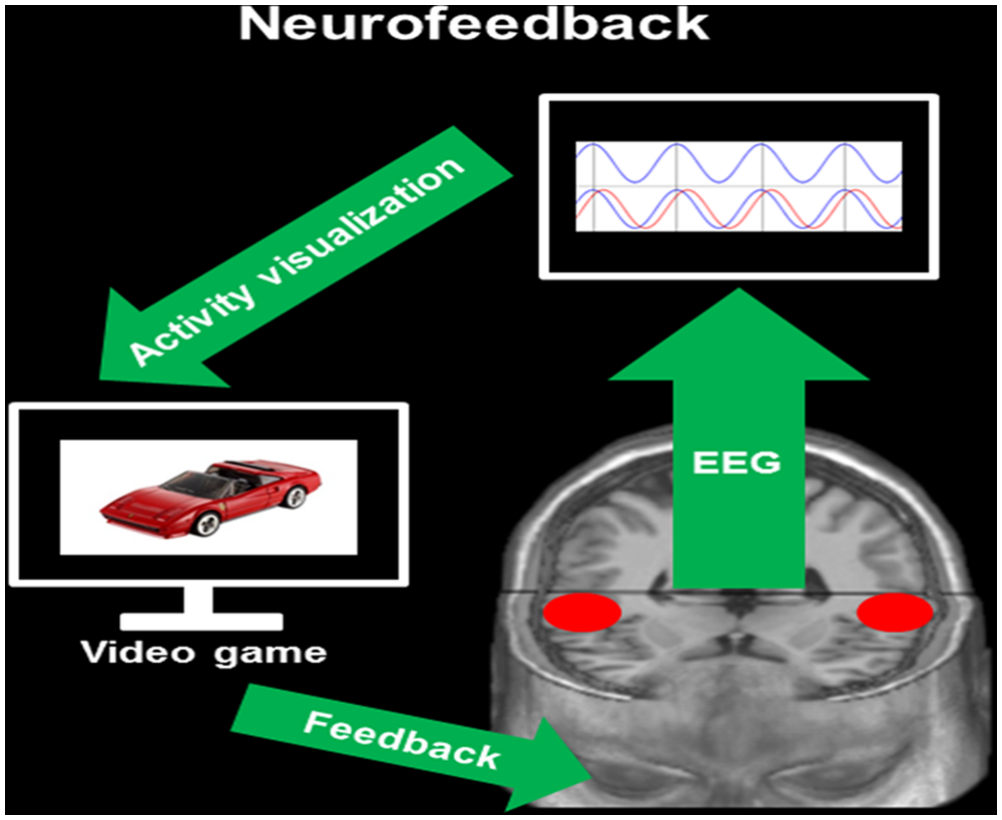
**Neurofeedback.** This figure provides a simplified overview of IFC-based neurofeedback. During EEG recording, the neurofeedback software provides information about the degree of phase alignment in *a priori* defined intracerebral ROIs [here the bilateral auditory-related cortex (ARC), red circles]. This information is visualized on a monitor by means of a brain–computer interface (activity visualization). Through the coupling of brain activity with a specific task (here video game), participants receive a visual feedback on the modulation of the own brain activity (feedback, here lagged phase synchronization).

Neurofeedback bases on cybernetic models consisting of using information about the physiological state of an organism for changing it in a specific direction (Gunkelman and Johnstone, 2005). Such cybernetic models can be utilized when the system to be analyzed is assumed to rely on closed signal-loops. This means that a change in a biological system (in this case brain activity) generates specific changes in the environment (in our case the feedback) that on his part triggers a modulation of the biological system (i.e., brain activity, see **Figure 2**). Due to the high temporal resolution of EEG as well as to novel mathematical applications, it is now possible to modulate the own brain activity in quasi real-time based on a specific feedback (i.e., visual, auditory, haptic, etc.). Meanwhile, there is a vast body of literature describing neurofeedback applications in several fields of clinical neuroscience (Schoenberg and David, 2014), including the treatment of addiction (Dehghani-Arani et al., 2013), attention-deficit hyperactivity disorder (Maurizio et al., 2014), depression (Young et al., 2014), epilepsy (Tan et al., 2009), and much more (Schoenberg and David, 2014).

An important prerequisite for clinical neurofeedback applications is to exactly know which intra- and extracerebral EEG parameters best reflect a specific natural or dysfunctional brain condition. In addition, an accurate identification of dysfunctional brain areas as well as of functional networks constitutes an important step toward evidence-based clinical applications. In the present article, we principally focus on IFC-guided neurofeedback rather than on the modulation of brain signals at the surface of the scalp. This line of argumentation is supported by the fact that scalp-signals are composed of miscellaneous and unspecific brain activity originating from a variety of brain regions. Furthermore, we are of the opinion that it is more efficient to dynamically change IFC between specific brain regions of interest instead of focusing on the modulation of restricted brain functions, than the former approach more likely takes into account the dynamic and interconnected nature of the human brain (see previous sections).

IFC guided neurofeedback applications base on exactly the same cybernetic models described in the previous section and depicted in **Figure 2**. However, in contrast to scalp-data based neurofedback, it is necessary to postulate clear assumptions on specific brain areas that are dysfunctional as well as on functional connectivity between these areas. Therefore, the first step of IFC-based neurofeedback is the identification of ROIs within a dysfunctional network. In addition, depending on the connectivity parameters to be trained (i.e., current density, phase synchronization, etc.) one should have a clear conception of the direction of modulations, that means increased or reduced intracerebral activity within the network of interest. In the case of IFC-based neurofeedback relying on the modulation of oscillatory phase alignment between different brain regions, specific knowledge about the relationships between brain functions and oscillations (i.e., delta, theta, alpha, beta, or gamma) is strictly required. In turn, we will describe the pathogenesis of a specific neurological disorder that is associated (at least in part) with auditory-related dysfunctions, namely developmental dyslexia. Based on a review of current research literature, we will propose concrete IFC-based neurofeedback applications relying on the entrainment of lagged phased synchronization. The modulation of lagged phase synchronization constitutes a fruitful approach in that this measure is supposed to reflect true connectivity by taking into account the delay of impulse propagation that is influenced by volume conduction (Pascual-Marqui et al., 2011). It is important to mention, that the ARC shows a huge inter-individual variability (Steinmetz et al., 1989; Zatorre, 2013) and asymmetry (Marie et al., 2013), and that this variability is additionally strongly influenced by training and expertise (Schlaug et al., 1995). Therefore, depending on the research question addressed and on the sample of subjects studied, it is important to take into account such influencing variables.

## DEVELOPMENTAL DYSLEXIA

The main purpose of the present work is to discuss concrete applications of IFC-based neurofeedback for the treatment of auditory-related dysfunctions. However, due to profound differences in the pathogenesis of such dysfunctions, here we will focus on developmental dyslexia only. It is important to mention that it is conceivable that similar approaches we present in association with dyslexia can be extended to other auditory-related dysfunctions, like for example tinnitus (Okamoto et al., 2010) or developmental language disorders (Heim et al., 2013).

Developmental dyslexia can be described as low reading and writing skills despite average intelligence, good educational support, and solid social background (Habib, 2000; Demonet et al., 2004). In the last three decades, several theories have been proposed for explaining the specific deficits in developmental dyslexia, including general perceptual/phonetic- (Tallal and Piercy, 1973; Merzenich et al., 1996; Stein, 2001; Goswami et al., 2002), attentional- (Bogon et al., 2014), and working memory deficits (Ahissar et al., 2006). Also visual (Lovegrove et al., 1980; Stein, 2012) and motor impairments (Nicolson and Fawcet, 1990) have been described.

Several of these theories postulate that developmental dyslexia is somehow related to auditory-related dysfunctions (Tallal and Piercy, 1973; Goswami et al., 2002). The “rapid processing deficit theory” proposed by Tallal and Piercy (1973) and Merzenich et al. (1996) postulates a specific impairment in the processing of fast-changing verbal cues, such as formant transitions and voice-onset time (VOT). In a similar way, Goswami et al. (2002) postulated that dyslexia is associated with a poor temporal resolution of speech sounds that specifically affects the processing of sound rise time. Other theories on dyslexia are rather centerd on phonological abilities (Stanovich, 1988; Serniclaes et al., 2001; Ramus, 2003; Ramus et al., 2013) and base on the assumption that dyslexic individuals are specifically impaired in building-up phonological representations (Stanovich, 1988). Finally, also impaired phonological awareness (Ramus, 2003; Ramus et al., 2013) and abnormal sensitivity to within phonemic category variations (Serniclaes et al., 2001) have previously been proposed to constitute the salient trait of developmental dyslexia. For a more comprehensive review of the literature on dyslexia, the reader is addressed to a previous work of Hamalainen et al. (2013).

Interestingly, most of the theories described above are compatible, at least in part, with the view that dyslexic children often show functional (Blau et al., 2009, 2010; Kast et al., 2011) and structural (Hugdahl et al., 2003; Brambati et al., 2004; Bloom et al., 2013) variations in the left ARC, a brain region that is relatively strongly involved in the processing of fast changing verbal and non-verbal cues and phonemes (Zatorre and Belin, 2001; Griffiths and Warren, 2002; Hickok and Poeppel, 2007). In addition, previous fMRI (Zatorre and Belin, 2001; Griffiths and Warren, 2002; Shaywitz et al., 2003, 2007; Hickok and Poeppel, 2007), DTI (Hoeft et al., 2011), and EEG (Dujardin et al., 2011) studies provided evidence for a stronger recruitment of right-sided ARC in dyslexic individuals during speech processing, possibly for compensating poor left-sided temporal resolution.

In a recent multi-pattern neuroimaging study Boets et al. (2013) reported intact phonetic representations (in terms of robustness and distinctness) in the bilateral ARC in adults suffering from dyslexia. Most notably, by combining functional and structural connectivity analyses, the same authors’ revealed reduced connectivity between bilateral auditory-related brain regions as well as between the auditory cortices and the left inferior frontal gyrus, the latter region being involved in higher order cognitive functions. These results are interesting in that they open the possibility to consider dyslexia as a neuropsychological state where not phoneme representation *per se*, but rather the access to these representations, is dysfunctional. A similar perspective can be taken into account when considering a recent publication of Vandermosten et al. (2013) where the authors combined DTI and EEG measurements in a sample of adult dyslexic individuals and found evidence for reduced white matter lateralization in the left posterior supratemporal plane and arcuate fasciculus. In addition, white matter lateralization in the posterior superior temporal gyrus and white matter integrity in the posterior part of the corpus callosum were related to phase coherence in bilateral auditory-related brain regions in the frequency range roughly corresponding to phonemic-rate modulations (∼20 Hz, β). Meanwhile, there is even evidence from longitudinal studies (Langer et al., 2013) showing that functional connectivity can change after only few weeks of training.

## INTRACEREBRAL CONNECTIVITY-GUIDED NEUROFEEDBACK AS A PUTATIVE REHABILITATIVE INTERVENTION FOR DEVELOPMENTAL DYSLEXIA

As described in the previous section, there is strong evidence showing dysfunctional left-sided (Rumsey et al., 1992; Temple, 2002) and compensatory right-sided (Shaywitz et al., 2003, 2007; Dujardin et al., 2011) brain activity in the ARC of dyslexic individuals. In addition, recent data point to altered functional (Poelmans et al., 2012; Vandermosten et al., 2013) and structural (Boets et al., 2013) connectivity among bilateral auditory-related brain regions as well as between the bilateral ARC and the left inferior frontal gyrus (Boets et al., 2013). The latter brain region is supposed to be involved in accessing higher-order phonological representations. With these previous results in mind, we will propose specific IFC-guided neurofeedback protocols that may be useful for ameliorating the auditory-related impairments often observed in dyslexic individuals. Please consider that these neurofeedback protocols are ordered in a hierarchical manner that means from small- to large-scale network reorganization.

### TRAINING PROTOCOL 1

**FIGURE 3:**
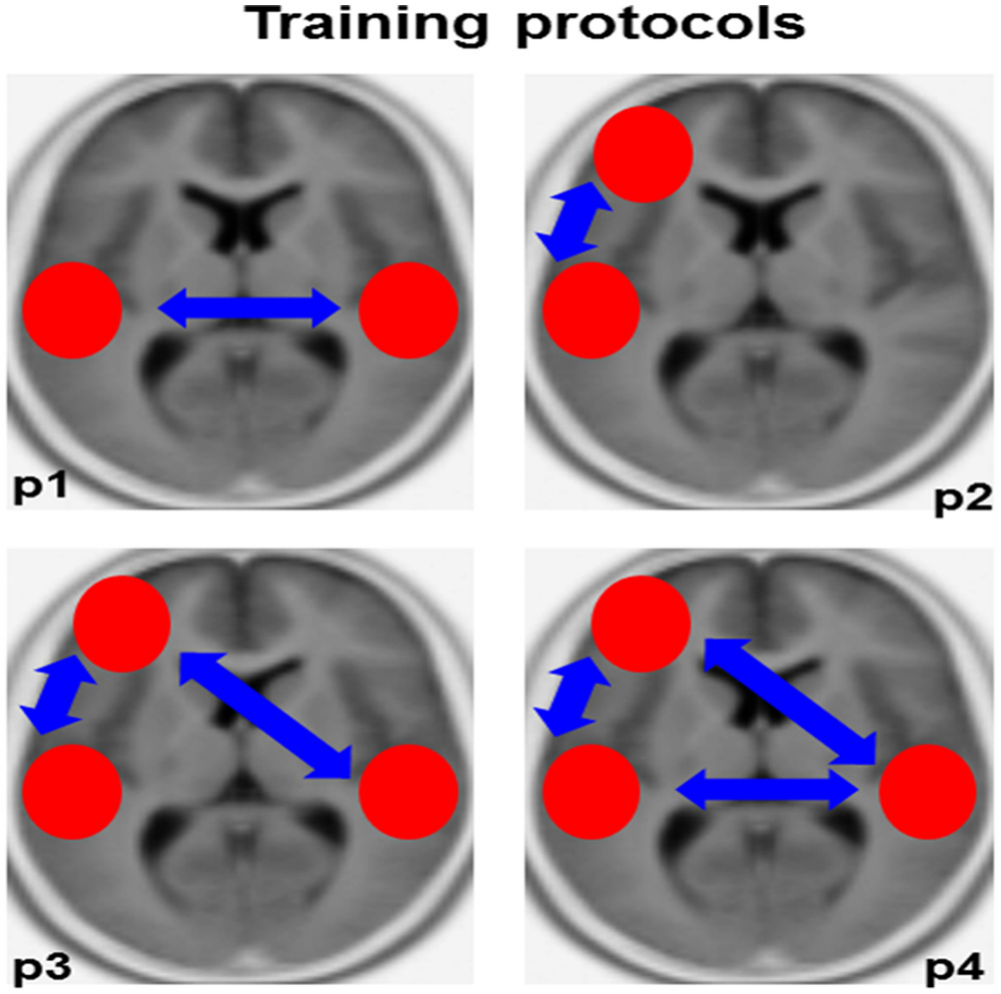
**Training protocols.** This figure provides an overview of the training protocols we propose for ameliorating auditory-related dysfunctions in dyslexic individuals. The red circles depict the intracerebral regions of interest, the blue arrows functional connectivity (lagged phase synchronization). P1–P4, protocol 1–4.

Based on the often observed hypoactivity of the left ARC in conjunction with the compensatory hyperactivity of its right-sided homolog in dyslexic individuals, we propose a training protocol targeting at ameliorating the division of labor (i.e., intracerebral lagged phase synchronization) between these two perisylvian brain regions (i.e., Brodmann areas 41/42/22). The reasoning beyond this training protocol is that the amelioration of functional connectivity between bilateral auditory-related brain regions may improve the functional capacity of the left ARC and at the same time reduce right-sided compensatory activity (**Figure 3**, p1). Along this vein, it is conceivable that an increase in phase alignment in at least two frequency bands may possibly improve reading skills, namely theta (∼4–7 Hz) and beta (∼13–20 Hz). In fact, theta oscillations roughly overlap with the processing rate of syllables, whereas beta oscillations coincide with the temporal dynamics of single phonemes (Poeppel, 2003; Poelmans et al., 2012). This reasoning is in line with previous work showing that dyslexic individuals are characterized by poor phonological awareness (Ramus, 2003; Ramus et al., 2013), a condition that is strongly dependent on the segmentation of single words into smaller units, namely syllables and phonemes.

Finally, it is important to mention that two previous studies of our group highlighted a relationship between the superiority of professional musicians (compared to non-musicians) in processing segmental speech cues (i.e., syllables varying in VOT and vowels) and functional (Kühnis et al., 2014) as well as structural (Elmer et al., accepted) connectivity among bilateral auditory-related brain regions. Based on these previous results, we believe that the improved and optimized auditory system of musicians can provide fruitful information for developing novel rehabilitative neurofeedback strategies targeting at optimizing auditory-related dysfunctions in dyslexic individuals. Certainly, future studies focusing on the validation of the training protocols we present in the present work are strictly required for optimizing rehabilitative power.

### TRAINING PROTOCOL 2

Previous work has described reduced functional and structural connectivity between the left ARC and the left inferior frontal gyrus in dyslexic individuals (Boets et al., 2013). Therefore, we may speculate whether in dyslexic individuals (probably mainly adults) not phoneme representation *per se*, but rather the access to these mnemonic representations in the left inferior frontal gyrus, is dysfunctional (Boets et al., 2013; Vandermosten et al., 2013). Based on previous work showing that neuronal oscillations in the theta-frequency range (∼ 4–7 Hz) reflect mnemonic processes (Kahana et al., 1999; Caplan et al., 2001; Ward, 2003; Sauseng et al., 2005; Elmer et al., under revision), information integration (Ward, 2003), and neuronal communication between distinct brain regions over long-range circuits (Ward, 2003; Polania et al., 2012), we propose the possibility to ameliorate the recruitment of higher order phonetic representations by increasing theta phase synchronization between the left ARC (BA 41/42/22) and the left inferior frontal gyrus (BA 44/45/47). See **Figure 3**, p2.

### TRAINING PROTOCOL 3

The third training protocol we propose here is an extension of “training protocol 2” (**Figure 3**, p3). Subjects are trained to increase intracerebral phase synchronization in the theta frequency range simultaneously between both the left and right ARC and the left inferior frontal gyrus (Boets et al., 2013).

### TRAINING PROTOCOL 4

This protocol implies a simultaneous combination of training protocols 1 and 3 (**Figure 3**, p4).

## FUTURE PERSPECTIVES

In the present opinion paper we discussed the possibility to ameliorate auditory-related dysfunctions by using IFC-based neurofeedback application targeting at changing the systemic functional brain organization rather than focusing on brain functions in isolation. It is important to mention that here we only addressed some putative application without any claim to completeness. In addition, we want to emphasize that future studies are strictly required for evaluating the rehabilitative relevance of the single training protocols we propose. We explicitly abstained from providing indications on specific training parameters (i.e., training duration and frequency) because we are of the opinion that neurofeedback therapists are best skilled for arranging and optimizing the training protocols we propose. Finally, it is important to remark that in our opinion a better understanding of simple connectivity circuits should be the first step. Only after having collected enough evidence for valid therapeutic applications in small-brain circuits, it makes sense to consider more systemic brain reorganization.

## CONNECTIVITY TOOLBOXES

For an overview of different neurofeedback applications, the reader is addressed to the following Wikipedia page: http://en.wikipedia.org/wiki/Comparison_of_neurofeedback_software

## Conflict of Interest Statement

The authors declare that the research was conducted in the absence of any commercial or financial relationships that could be construed as a potential conflict of interest.
